# Rolling Stones Management of Gallstone Ileus Through a Single-Institution Retrospective Analysis

**DOI:** 10.7759/cureus.93670

**Published:** 2025-10-01

**Authors:** Carla M Cruz Rocha, David J Alvarez Chavez, Maria C Torres Gonzalez, Carlos Alfredo Bautista Lopez, Carlos E Perez Tristan, Heriberto Godínez Serrato, Esther A Casado de la Torre, Yael E Silva García, Gadiel A Cruz Rocha, Jaime Gonzalez Hernadez

**Affiliations:** 1 General Surgery, Universidad de Guadalajara, Hospital Civil de Guadalajara "Juan I Menchaca", Guadalajara, MEX

**Keywords:** biliary digestive fistulas, gallstone, gallstone ileus, intestinal obstruction, rigler's triad

## Abstract

Biliodigestive fistulas are a complication of gastrointestinal pathologies, often arising from chronic recurrent cholecystitis. These fistulas disrupt normal biliary physiology, leading to bile reflux or the entry of intestinal contents into the biliary system, with gallstone ileus as a significant consequence. This condition typically results in mechanical bowel obstruction due to the migration of gallstones into the intestinal lumen. Diagnosis can be challenging due to nonspecific symptoms, and surgical intervention is often required. This retrospective study reviews cases of gallstone ileus at a single institution, focusing on clinical presentation, diagnostic methods, surgical approaches, and outcomes. Eight patients, all elderly females with an average age of 77 years, presented with symptoms such as abdominal pain, vomiting, and absence of evacuation. Imaging played a crucial role in diagnosis, and surgical management was primarily enterolithotomy. Mortality was notable, with comorbidities contributing to poor outcomes. The findings suggest that a personalized, staged surgical approach may be beneficial, particularly for high-risk patients. This study highlights the importance of considering biliodigestive fistulas in the differential diagnosis of intestinal obstruction and the need for further research into optimal treatment strategies.

## Introduction

Biliary digestive fistulas are rare complications arising from acute gastrointestinal pathologies, with chronic recurrent cholecystitis being the primary cause [1]. Other notable etiologies include perforation of duodenal ulcers into the biliary tree and neoplastic infiltration originating from the biliary or gastrointestinal tract. Less frequently, conditions such as echinococcus cysts, liver or renal abscesses, Caroli’s disease, penetrating trauma, and necrotic colitis can also lead to this abnormal communication [2]. 

The establishment of a biliodigestive fistula disrupts normal biliary physiology, creating a pressure gradient between the bowel and biliary tree. This imbalance can result in bile reflux into the duodenum or the entry of intestinal contents into the biliary system, due to the lack of protective mechanisms typically provided by Oddi’s sphincter and the anatomical configuration of the common bile duct [2,3]. 

Gallstone ileus, a rare but significant complication, accounts for approximately 1% to 4% of all cases of mechanical small bowel obstruction [4]. This condition manifests as a mechanical bowel obstruction, triggered by the migration of gallstones from the biliary tree into the intestinal lumen through a bilioenteric fistula [5]. As these gallstones traverse the bowel, they often increase in size from the addition of intestinal contents, ultimately leading to obstruction in the ileum when their diameter exceeds 2.5 cm [6].

The diagnosis of gallstone ileus can be challenging due to its non-specific presentation, which often includes nausea, vomiting, colicky pain, abdominal distension, and discomfort in the upper right quadrant. Surgical intervention is typically required to alleviate the obstruction, as spontaneous passage of the stones is uncommon, especially when presented proximal to the ileocecal valve [7]. However, there is ongoing debate regarding the optimal surgical technique, with enterolithotomy via laparotomy frequently cited as a safe and effective approach, particularly for patients with comorbidities or hemodynamic instability. This study aims to underscore the significance of tailored surgical strategies, taking into account the unique clinical circumstances of each patient [1,8].

## Materials and methods

Study design

A retrospective, observational, and descriptive study was conducted at the Civil Hospital of Guadalajara Juan I. Menchaca. It included patients diagnosed with intestinal obstruction secondary to gallstone ileus between January 2021 and August 2024.

Participant selection

Patients included in this study were those who presented with intestinal obstruction attributable to gallstone ileus, confirmed by the presence of at least one gallstone visible on computed tomography (CT) imaging. Data were collected for all eligible cases identified during the study period.

Data collection

Patient demographics, including age, sex, and comorbidities, were systematically recorded. All data were anonymized to maintain patient confidentiality. Clinical data included the mean time from symptom onset to diagnosis, length of hospital stay, clinical presentation, operative findings, and postoperative outcomes. Specific attention was given to imaging studies and biochemical tests performed before surgical intervention.

Additional information was gathered regarding the type of surgical management employed, the location of the obstructing gallstone, and any complications or mortality associated with the intervention. Postoperative follow-up data were also collected to assess long-term outcomes.

Ethical considerations

This study was approved by the institutional review boards (IRB) of the participating sites. Given the retrospective nature of the study and the minimal risk involved, informed consent was not required as per the IRB's determination.

## Results

We identified a total of eight patients with biliary ileus in our institution (Table 1).

**Table 1:** Summary of cases, characteristics, epidemiological, clinical, complementary tests and treatment.

Patient	Age (years)	Days of Symptoms	White Blood Cells (10 ^9^/L​​​)	CT Rigler Triad	American Society of Anesthesiologists (ASA) Physical Status Classification System	Procedure	Obstruction Area	Days of Hospital Stay	Complication
1	52	5	11.2	YES	I	ENTEROLITHOTOMY	SMALL INTESTINE 140 CM FROM ILEOCECAL VALVE	11	NO COMPLICATIONS
2	88	15	15.3	YES	III	ENTEROLITHOTOMY	SMALL INTESTINE 220 CM FROM THE ANGLE OF TREITZ	11	DEATH
3	93	2	17.9	YES	II	ENTEROLITHOTOMY	SMALL INTESTINE 40 CM DISTANCE FROM THE ILEOCECAL VALVE	9	NO COMPLICATIONS
4	59	6	7.4	NO	III	ENTEROLITHOTOMY WITH CHOLECYSTECTOMY AND FISTULA REPAIR	SMALL INTESTINE 40 CM AND 120 CM OF THE ILEOCECAL VALVE	22	NO COMPLICATIONS
5	65	3	6.4	YES	I	ENTEROLITHOTOMY	SMALL INTESTINE 300 CM FROM THE ANGLE OF TREITZ	5	NO COMPLICATIONS
6	93	10	11.2	YES	III	NO SURGERY WAS PERFORMED	LARGE INTESTINE (CECUM)	9	DEATH
7	97	9	15.5	YES	III	ENTEROLITHOTOMY	SMALL INTESTINE 70 CM FROM THE ILEOCECAL VALVE	9	DEATH
8	73	8	15.4	YES	III	NO SURGERY WAS PERFORMED	LARGE INTESTINE	17	DEATH
Average	77.5	7.25	12.5					11.6	

**Figure 1:**
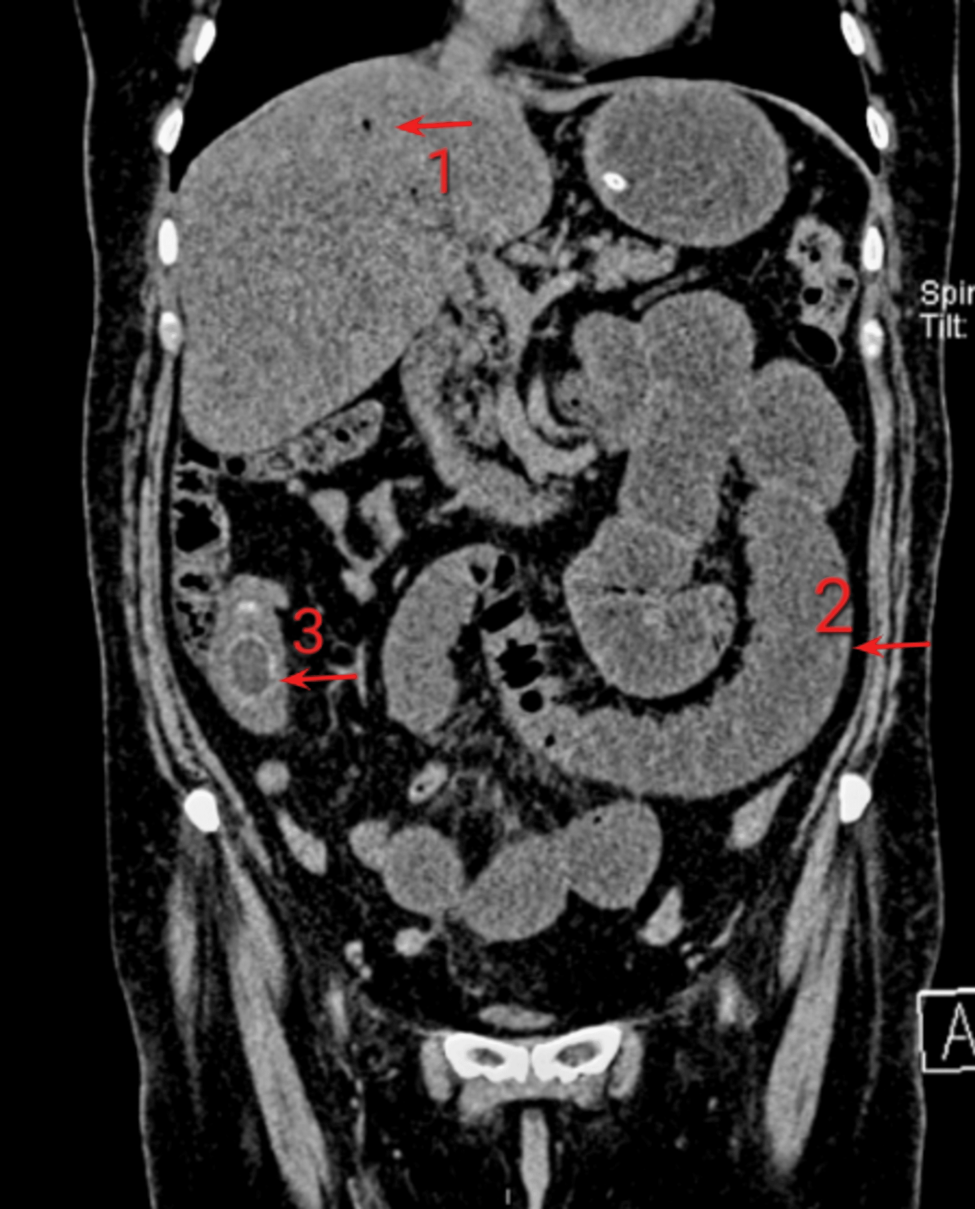
Rigler's Triad. 1) Pneumobilia, 2) Small bowel obstruction, 3) Ectopic gallstone

According to the literature, gallstone ileus tends to affect predominantly female and older adult patients, often with significant comorbidities [1,4,7,8]. This was consistent with our cohort, composed entirely of female patients with a mean age of 77.5 years. The clinical presentation was nonspecific, including diffuse abdominal pain, bilious vomiting, and constipation, findings commonly seen in other forms of small bowel obstruction [8-10]. This lack of specificity frequently leads to diagnostic delay; in our series, the mean symptom duration before hospitalization was 7.25 days. At present, no specific biomarkers exist for the diagnosis of gallstone ileus. However, in our institution, metabolic alterations such as elevated creatinine, hypercalcemia, and hyponatremia were frequently observed. Radiologically, Rigler’s triad comprises aerobilia, intraluminal gallstone, and evidence of intestinal obstruction (Figure 1). This remains a classical diagnostic hallmark, although its sensitivity is limited [10-12].

It can be appreciated in a simple abdominal X-ray or computed tomography. In our institution, seven (87.5%) of the patients presented this triad. The CT imaging confirmed the diagnosis and distinguished the components and level of disruption. Having intraoperative findings in most cases that were surgically intervened, there was an obstruction in the small intestine in six (75%) of the patients, specifically in the terminal ileum. The mean length of stay was 11.6 days. With a mortality of four of the eight cases reported (50%), all four of these patients presented a high surgical risk (American Society of Anesthesiologists (ASA) III: a patient with severe systemic disease). Table 2 shows the relationship between ASA physical status classification system and mortality.

**Table 2:** Relationship between American Society of Anesthesiologists (ASA) physical status classification system and mortality.

American Society of Anesthesiologists (ASA) Physical Status Classification System	Cases	Mortality
ASA I - II	3	0
ASA III-IV	5	4

**Figure 2:**
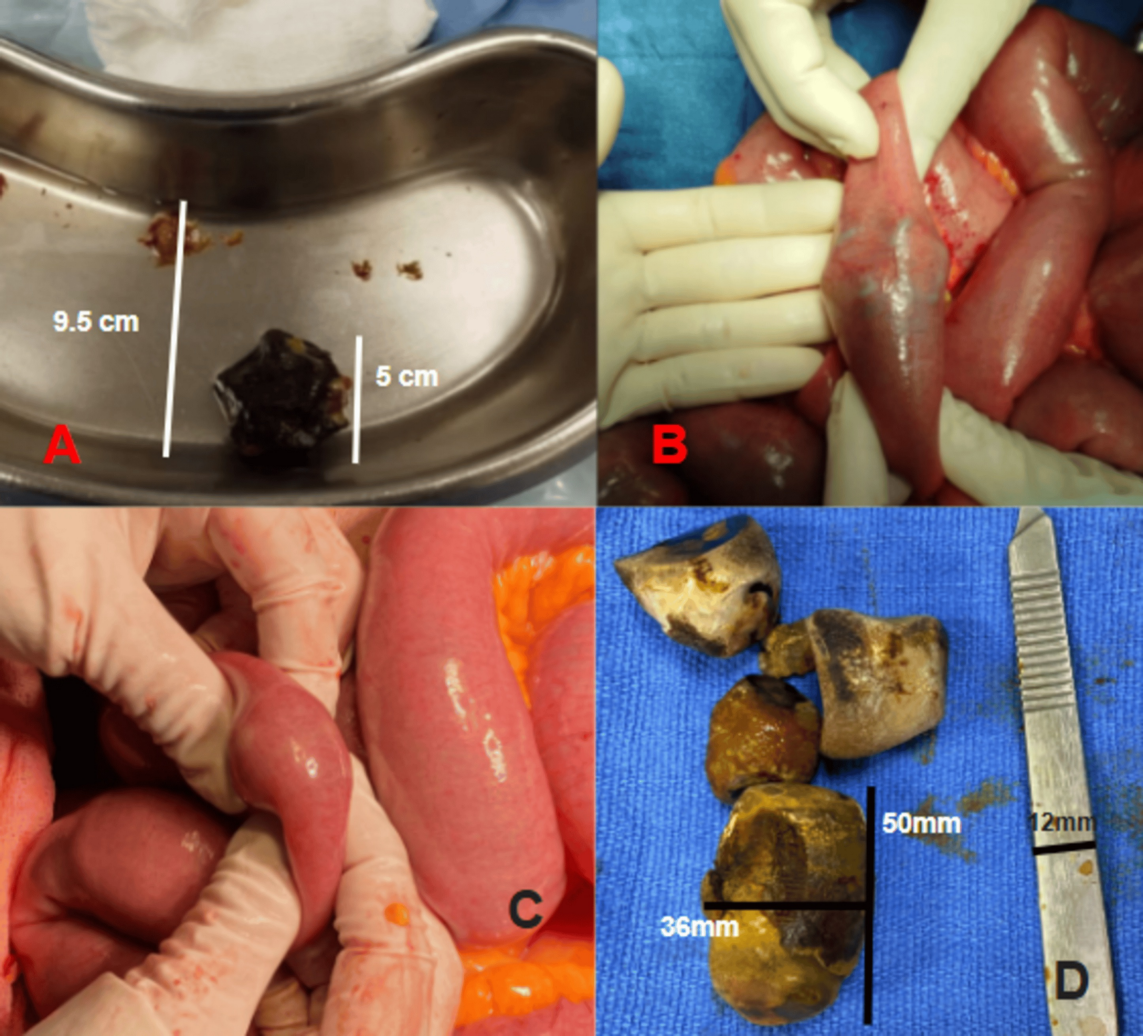
Gallstone ileus cases. A) Spiculated gallstone measuring 5 cm. B) Gallstone ileus prior to enterolithotomy. C) Multiple gallstone ileus prior to enterolithotomy. D) Multiple gallstones measuring 50 mm to 36 mm.

The surgical approach was determined by gallstone location and preoperative surgical risk. Enterolithotomy with primary closure (one-stage procedure) was the most common management, performed in five patients (62.5%). Intraoperative findings revealed gallstones in the terminal ileum in six patients (75%). The mortality rate associated with enterolithotomy was 40%, mainly in patients with high surgical risk (Figure 2).

**Figure 3:**
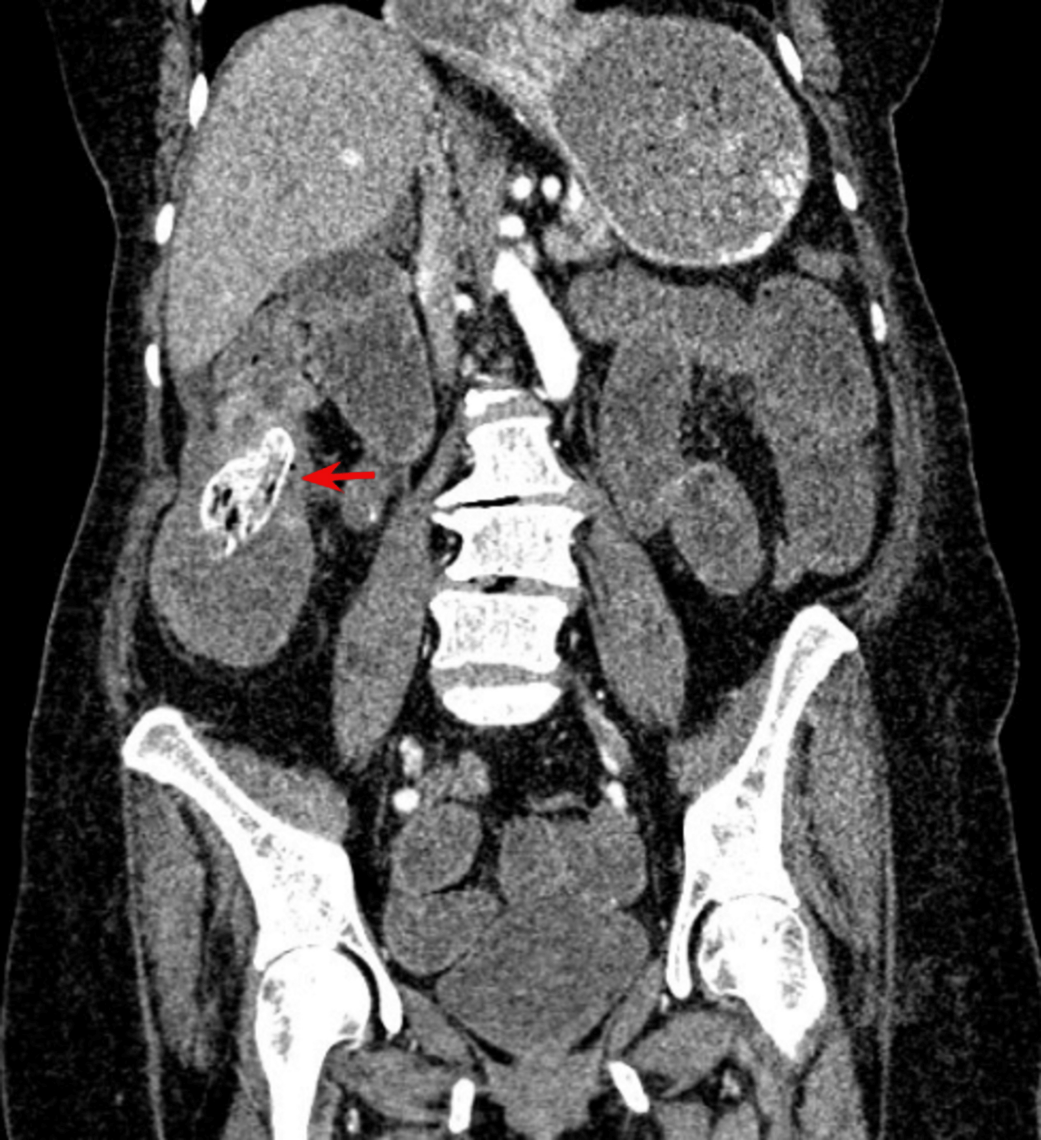
Ectopic gallstone in caceum

Two of our patients were not intervened due to the site of the gallstone being in the colon, since due to the location of the stone it is likely that they will be expelled spontaneously (Figure 3).

**Figure 4:**
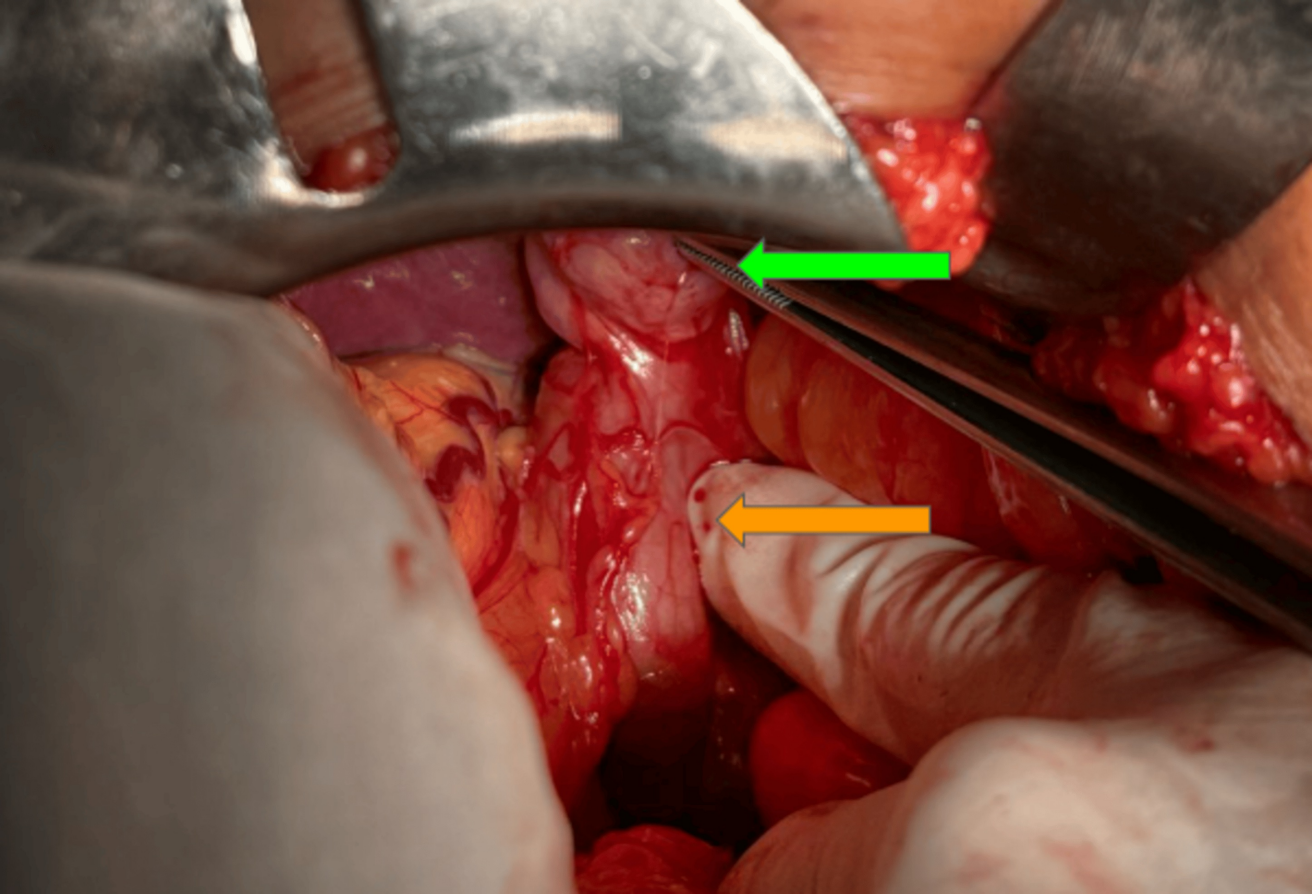
Bilioenteric fistula. Green arrow: gallblader; Orange arrow: duodenum

In one of our patients, a single-stage intervention (enterolithotomy with cholecystectomy and fistula repair) was successfully performed, leading to a positive outcome, largely due to the patient’s younger age and low surgical risk (Figure 4).

The choice of surgical approach appears to be closely linked to patient outcomes and mortality as depicted in Table 3.

**Table 3:** Relationship between type of surgical management vs mortality.

Type of Surgery	Number of Cases	Mortality
Enterolithotomy	5	2
Single Stage Definitive Surgery	1	0
No Surgery Was Performed	2	2

## Discussion

Gallstone ileus represents approximately 4% of all causes of intestinal obstruction in the general population, but its incidence rises up to 25% among patients older than 65 years [4]. Consequently, most of the available publications correspond to isolated case reports.

In recent years, its frequency appears to be increasing, being reported as up to seven times more common in individuals over 70 years of age [9]. Gallstone ileus remains an uncommon etiology of mechanical small bowel obstruction, generally reported in less than 5% of cases [1,9]. The slight variation in reported incidence (4% vs. <5%) likely reflects differences in study populations and methodological approaches across epidemiological reports.

This condition predominantly affects elderly female patients who usually present with other significant comorbidities [5,7]. The obstruction results from the impaction of a gallstone within the intestinal lumen, most frequently in the ileum, with the usual route of entry being through a biliary-enteric fistula [2,6]. A possible explanation for its increased incidence in older adults may be related to the higher prevalence of gallstone disease, delayed diagnosis due to nonspecific symptoms, and the greater likelihood of fistula formation in long-standing cholelithiasis. This is a rare complication of cholelithiasis occurring in less than 3% of all cases [10].

The pathophysiology of the fistula is linked to the Mirizzi syndrome, this being the hepatic duct obstruction caused by an extrinsic compression from an impacted stone in the cystic duct. The pericholecystic inflammation after cholecystitis leads to development of adhesions between the biliary tract and the enteric system. The pressure and necrosis of the gallstones against the biliary wall cause erosion and fistula formation. This compression is the genesis for the formation of a fistula acting as an exit route for the gallstone [11]. The bilioenteric fistula has common locations: cholecystoduodenal, cholecystocolic, and cholecystogastric. The most common is the cholecystoduodenal fistula with 60% of the cases reported. The gallstones that are large enough to cause an obstruction are normally greater than 2.5 cm in diameter [6].

The diagnosis is a challenge due to the low rate of cases, as well as the intermittent and low specificity of the symptoms. The patient normally refers to diffuse abdominal pain and vomiting. The obstruction may be intermittent as a result of the stone passing through the bowel lumen. The mean intermittent symptoms may be present five days before the hospital admission. The average symptom duration before hospital admission in our institution was seven days [9]. On physical examination, the patients normally present dehydration, with abdominal distension and increased bowel sounds. Jaundice is uncommon due to the presence of an exit route for the biliary juices [9,10]. 

Biochemical abnormalities most common were leukocytosis, electrolyte imbalance due to dehydration [11]. Currently, the confirmatory diagnosis can be made through a CT scan. Previously, the diagnosis was made through X-ray films or ultrasound, with the only confirmatory diagnosis being through surgery. The visualization of the gallstone on X-ray films is low since most stones are radiolucent and the bowel gas or bone structures obscure the gallstones. A CT scan not only helps with the diagnosis but also identifies the location of the bowel obstruction and looks for any other complications [12,13]. The signs presented in the CT scan are gallbladder wall thickening, neumobilia, intestinal obstruction, and obstructing gallstones. Rigler's triad is the appearance on plain radiograph of pneumobilia and intestinal obstruction by an obstructing gallstone [14]. 

As for comorbidities, many affected patients have serious concomitant medical illnesses, including coronary disease, pulmonary disease, and poorly controlled diabetes mellitus. In our institution, the patients were not only elderly with a mean age of 77 years, but they also presented comorbidities such as pneumonia and poorly controlled diabetes [8,9]. 

The gallstone ileus involves three common surgical pathologies: cholelithiasis, biliary-enteric fistula, and intestinal obstruction. The treatment is primarily surgical. Addressing the intestinal obstruction through enterotomy with removal of the gallstone. Cholelithiasis and biliary-enteric fistula are addressed together with a combined biliary procedure involving cholecystectomy and fistula closure. Currently the procedure consists of the cholecystectomy and the fistula closure. These stages can be performed in a single surgery or multiple surgeries [15-17]. 

Multiple syndromes have been described depending on the site of obstruction and clinical presentation. Bouveret’s syndrome is a proximal variant characterized by gastric outlet or duodenal obstruction secondary to a gallstone migrating through a cholecystoduodenal fistula [9]. Barnard’s syndrome occurs when the stone becomes impacted at the ileocecal valve. Karewsky’s syndrome represents the chronic form of gallstone ileus, defined by recurrent episodes of abdominal pain caused by repeated migration of gallstones through the intestine [4,5,14,18].

The first-line surgical procedure is enterolithotomy, most commonly performed via laparotomy. A longitudinal enterotomy is made on the antimesenteric border proximal to the point of impaction, followed by proximal “milking” of the stone and extraction. The enterotomy is then closed transversely to avoid luminal narrowing (Heineke-Mikulicz technique) [19-21]. The entire small bowel must be carefully explored to rule out additional gallstones. Although laparoscopic enterolithotomy has been described, conversion to open surgery is frequent due to the technical challenge of evaluating a dilated bowel and locating the obstructing stone. Bowel resection is indicated in cases of perforation, severe ischemia, necrosis, or when the gallstone cannot be mobilized [19-24].

Management of the biliary fistula and gallbladder can be performed either in a single-stage surgery (enterolithotomy with cholecystectomy and fistula repair) or a two-stage procedure. The single-stage approach is more radical and generally reserved for younger patients with fewer comorbidities, as it is associated with longer operative time and higher postoperative morbidity. However, it has the advantage of definitively treating the underlying cause, thereby preventing recurrent gallstone ileus, persistent biliary-enteric fistula, cholecystitis, cholangitis, and even gallbladder carcinoma [14].

In contrast, the two-stage strategy - initial enterolithotomy followed later by elective cholecystectomy and fistula repair - offers reduced operative risk, shorter surgical time, and allows stabilization of critically ill patients before definitive management [15]. In high-risk patients, definitive biliary surgery may be deferred or omitted, since spontaneous closure of the fistula occurs in up to 50% of cases, particularly when the cystic duct remains patent and no residual gallstones are present [11,16].

Limitations

This study is limited by its retrospective, single-center design and small sample size, which reduce the generalizability of findings. Variability in diagnostic methods, particularly the transition to CT scans, may have influenced accuracy. The high prevalence of comorbidities in elderly patients complicates outcome assessment, and the observed mortality rate reflects the challenges of managing this condition in high-risk individuals. Additionally, delays in diagnosis due to nonspecific symptoms remain a critical issue. Further prospective studies with larger cohorts are needed to optimize diagnostic and surgical strategies.

## Conclusions

In conclusion, our study highlights the challenges of managing gallstone ileus and underscores the need for a personalized treatment approach. Enterolithotomy remains a safe and effective option, particularly in high-risk patients, where a two-stage strategy may minimize surgical risks. Given the variability in surgical techniques, further research is needed to establish standardized guidelines. Additionally, recognizing biliodigestive fistulas in the differential diagnosis can improve patient outcomes. By sharing our experiences and insights, we hope to contribute to the ongoing discourse in this field and encourage future investigations aimed at refining treatment strategies for gallstone ileus.
